# CLARINET: efficient learning of dynamic network models from literature

**DOI:** 10.1093/bioadv/vbab006

**Published:** 2021-06-03

**Authors:** Yasmine Ahmed, Cheryl A Telmer, Natasa Miskov-Zivanov

**Affiliations:** 1 Electrical and Computer Engineering Department, University of Pittsburgh, Pittsburgh, PA 15213, USA; 2 Department of Biological Sciences, Carnegie Mellon University, Pittsburgh, PA 15213, USA; 3 Bioengineering Department Computational and Systems Biology Department, University of Pittsburgh, Pittsburgh, PA 15213, USA

## Abstract

**Motivation:**

Creating or extending computational models of complex systems, such as intra- and intercellular biological networks, is a time and labor-intensive task, often limited by the knowledge and experience of modelers. Automating this process would enable rapid, consistent, comprehensive and robust analysis and understanding of complex systems.

**Results:**

In this work, we present CLARINET (**CLARI**fying **NET**works), a novel methodology and a tool for automatically expanding models using the information extracted from the literature by machine reading. CLARINET creates collaboration graphs from the extracted events and uses several novel metrics for evaluating these events individually, in pairs, and in groups. These metrics are based on the frequency of occurrence and co-occurrence of events in literature, and their connectivity to the baseline model. We tested how well CLARINET can reproduce manually built and curated models, when provided with varying amount of information in the baseline model and in the machine reading output. Our results show that CLARINET can recover all relevant interactions that are present in the reading output and it automatically reconstructs manually built models with average recall of 80% and average precision of 70%. CLARINET is highly scalable, its average runtime is at the order of ten seconds when processing several thousand interactions, outperforming other similar methods.

**Availability and implementation:**

The data underlying this article are available in Bitbucket at https://bitbucket.org/biodesignlab/clarinet/src/master/

**Supplementary information:**

Supplementary data are available at *Bioinformatics Advances* online.

## 1 Introduction

Computational modeling has an important role in the process of explaining complex systems. It allows for capturing their dynamics, it helps identify gaps in our understanding and thus, often leads to new questions and the search for missing information (Epstein, 2008). In biology, model creation is often highly dependent on human input, it requires reading hundreds of papers to extract useful information, incorporating background and common-sense knowledge of domain experts and conducting wet lab experiments (Fisher and Henzinger, 2007). Moreover, the amount of biological data is constantly growing, further augmenting the issues of data inconsistency and fragmentation. Therefore, automating the process of creation and extension of models is critical for consistent, comprehensive and reproducible studies of biological systems.

Mechanistic models have been used to explain how biomolecular signaling pathways regulate cell functions. Usually, modelers start with a few seed components and their interactions to build a baseline model, which summarizes the modeler’s knowledge about the system. Depending on the questions to be answered by the model, the baseline model is often further extended with the information extracted from literature or obtained from the domain experts (Miskov-Zivanov, 2015). Several machine reading engines have been developed recently focusing on biomedical literature and extracting hundreds of thousands of events from thousands of papers within hours (Valenzuela-Escárcega *et al.*, 2017). In order to add this information to existing models, or to build new models from it, one needs methods and tools for systematic selection of the most useful information from this large machine reading output.

The biomolecular signaling pathway models can be assembled using the INDRA tool (Gyori *et al.*, 2017), which relies on collecting and scoring new information extracted either from literature by natural language processing algorithms or from pathway databases. To select the most valuable information, each statement is evaluated, and its overall belief score is computed as the joint probability of correctness implied by the evidence. On the other hand, another tool, FLUTE (Holtzapple *et al.*, 2020), has been recently proposed to further filter the extracted interactions using public databases in order to eliminate incorrect or nonrelevant information extracted by machine readers. FLUTE also allows users to select confidence thresholds for interactions. While INDRA and FLUTE are powerful automated tools that can be used to extract and filter useful information from literature, they do not automatically extend existing models with new information.

In Liang *et al.* (2017), the authors proposed a method that starts with a baseline model and systematically selects interactions that were automatically extracted from published papers. The goal of Liang *et al.* (2017) was to build a model that satisfies desired system properties or to identify new therapeutic targets. As results in Liang *et al.* (2017) demonstrate, automatic model extension is a promising approach for accelerating modeling, and consequently, disease treatment design. The authors in Liang *et al.* (2017) organize the information extracted from literature into layers, based on their proximity to the baseline model. Another extension method that uses genetic algorithm has been proposed recently in Sayed *et al.* (2018a). The genetic algorithm-based approach was able to select a set of extensions that led to the desired behavior of the final expanded model. The disadvantages of the genetic algorithm-based approach include nondeterminism, as the solution may vary across multiple algorithm executions on the same inputs, and a significant increase in runtime with an increase in the size of the model and in the amount of new extracted information.

In this work, we propose a novel method and a tool, CLARINET (**CLAR**Ifying **NET**works) that automatically and efficiently extends existing models, by selecting the most relevant and useful information. When compared with the work in Liang *et al.* (2017), CLARINET is more practical as it provides connected set of extension events that are at the same time connected to the baseline model. In contrast to the genetic algorithm-based method (Sayed *et al.*, 2018a), CLARINET is more efficient and provides deterministic solutions. Therefore, the main contributions of the work presented here include:

1. An automated, fast methodology and a tool that utilizes the knowledge published in the literature and suggests model extensions.
2. A novel approach to study events extracted from literature as a collaboration graph, including several metrics that rely on the event occurrence and co-occurrence frequency in literature.
3. A parametrizable tool that allows users to explore different selection criteria, when automatically identifying the best extensions for their models.

## 2 Clarinet inputs

CLARINET has two inputs, a machine reading output of selected literature, and a baseline model that will be extended with the information from the reader output. In this section, we provide a brief description of the kind of information that is extracted from literature and the type of baseline models that CLARINET can extend.

### 2.1 Information extraction from literature

The biomedical literature reading engines (Burns et al., 2016; Valenzuela-Escárcega *et al.*, 2017) are capable of extracting hundreds of thousands of cell signaling events from thousands of papers, in a few hours. As part of these events, the reading engines identify entities, i.e. the participants of biochemical reactions, usually proteins, chemicals, genes or even biological processes. For each extracted entity, reading engines provide its name, the unique standard identifier (ID) found in public databases [e.g. UniProt (Bateman *et al.*, 2017), GO (Ashburner *et al.*, 2000), and HMDB (Wishart *et al.*, 2018)] and the entity type. The extracted events usually represent interactions between the entities, that is, various intracellular events, such as mechanisms of post-translational modification (e.g. binding, phosphorylation, ubiquitination, etc.), transcription, translation, translocation, as well as qualitative events of increasing or decreasing amount or activity. Besides the entity and event information, machine reading also provides the event evidence, the published paper and the sentence from which the event was extracted.

**Fig. 1.**
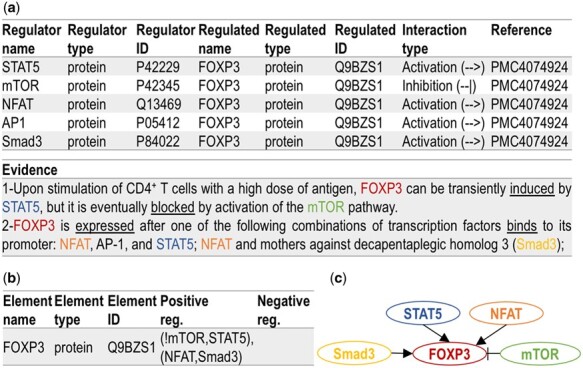
CLARINET inputs: (**a**) example of five events extracted by reading engines, represented in a tabular form; (**b**) model element FOXP3, and its influence set (positive and negative regulators), represented in a tabular BioRECIPES format, where ‘!’ is a logical NOT, ‘()’ are used for logical AND and ‘,’ is a logical OR [the influence set notation is described in Sayed *et al.* (2018c)] (**c**) graphical representation of FOXP3 and its influence set

In Figure 1a, we show, using a tabular format, the main components of five entities in a typical machine reading output (Valenzuela-Escárcega *et al.*, 2017) obtained from two sentences (listed under Evidence). Each row of the machine reading output represents one extracted event, a directed signed interaction between regulator entity and regulated entity. It is worth noting that even the state-of-the-art machine reading engines still output some number of erroneous events, or events that are missing entities, or events that include interactions not useful for modeling, and therefore, event filtering methods often need to be applied (Holtzapple *et al.*, 2020). Furthermore, not all extracted events can be used as model extensions, as some are just corroborating or even contradicting models. Since filtering and classifying machine reading output is beyond the scope of the work presented here, in the rest of this paper, we will assume that the extracted events have already been filtered and classified, and we will use only the potential model extensions, the event set that we will refer to as *Extracted Event Set* (*EES*). As the same event can be extracted from many different papers, we define *N* as a total number of events in EES, and *M* as a number of distinct events in EES (*N* ≥ *M*).

### 2.2 Dynamic and causal network models

The underlying static structure of a dynamic or a causal network model of intracellular signaling can be described as a directed graph G(V, E), with a set of nodes V and a set of edges E (Zañudo *et al.*, 2018). Each node *v ∈ V* corresponds to one model element, and each edge *e*(*v_i_*, *v_j_*) ∈ E represents a directed interaction in which node *v_i_*, is a regulating element, and node *v_j_* is a regulated element. Here, we will refer to the set of positive and negative regulators of an element (activators and inhibitors, respectively) as its influence set. Besides their network structure, dynamic models also contain update functions that are used to change states of model elements, and thus, enable simulation of model element behavior in time (Albert et al., 2008; Sayed *et al.*, 2018b).

To represent all the details of a model, including its network structure and the update functions, we use the BioRECIPES tabular element-based format proposed in Sayed *et al.* (2018c), as it is able to capture all the relevant information for dynamic and causal modeling. An example of a model element (FOXP3) and its influence set represented using the BioRECIPES format is shown in Figure 1b. The BioRECIPES format includes a number of element and influence set attributes, such as name, type (protein, gene or a chemical), identifier from a database [e.g. UniProt (Bateman *et al.*, 2017)], variable that represents the element state, all regulators in the influence set (including the notation used for update functions, as seen in Figure 1(b)), and evidence statements with the text from which the event was obtained (if any).

We note here that both ‘entities’ in machine reading output described in Section 2.1, and ‘elements’ in the models that we study correspond to components of biological systems, and that both the literature extracted ‘events’ and the ‘interactions’ between model elements correspond to biological interactions (e.g. biochemical reactions). In the rest of this paper, we will represent events/interactions using graphs and refer to them as ‘edges’ (when discussing graphs) or ‘events’ (when discussing models or EES). For example, the events extracted from the sentences in Figure 1a and combined into an influence set of FOXP3 in Figure 1b, are shown as a graph in Figure 1c. We note here that these directed graphs are different from the undirected event collaboration graphs that will be discussed in detail in Section 3.1.

## 3 Clarinet methodology

**Fig. 2.**
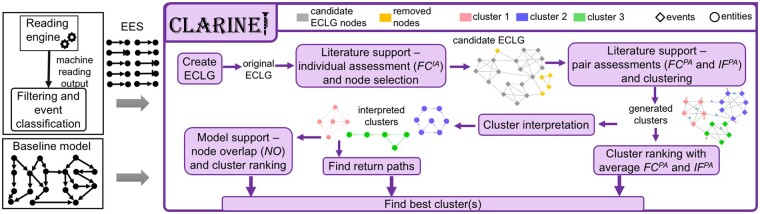
Illustration of CLARINET framework: (Left) CLARINET inputs: EES and Baseline model. (Right) Flow diagram of the CLARINET processing steps and outputs

In this section, we describe the main steps and components of the CLARINET methodology, which are also outlined in Figure 2.

### 3.1 Event collaboration graph creation and individual assessment

Following the notion of collaboration graphs that are often used to model social networks (Grossman and Ion, 1995), we introduce the *event collaboration graph* (*ECLG*). In social networks, nodes represent participants and edges connect pairs of nodes that have collaborative relationships. Similarly, we define an ECLG as a weighted undirected graph G (V, E, Wv, We), where V is a set of graph nodes, each representing a distinct event extracted from literature, E is a set of graph edges, each edge indicating a co-occurrence in the same paper of the two events corresponding to its adjacent nodes. Wv and We are sets of node and edge weights, respectively.

We will refer to the ECLG created from the EES as an *original ECLG*. We compute the weights Wv and We based on the frequency of the event occurrence and co-occurrence in the EES using several metrics proposed in this and the following subsection.

#### 3.1.1 Individual assessment (IA)

As a measure of the frequency of occurrence, within EES, of each distinct event that belongs to ECLG, we propose a *frequency class* (*FC*) metric. This metric is similar to the frequency class metric in computational linguistic (also called Häufigkeitsklasse), which measures word frequency in a corpus of words (Brown *et al.*, 1992; Weeber *et al.*, 2000) and has a number of uses and effects. In this work, we will use frequency class to identify the most and the least frequent events. We will show later in Section 5 that CLARINET is able to select the most relevant events in an accurate way using the frequency class metric. Instead of computing *FC* for words in a text, given the EES (with *N* total events and *M* distinct events), we compute frequency class ${FC}_{i}^{IA}$, for each extracted distinct event, *i* = 1,..,*M*:

(1) $${FC}_{i}^{IA}=\left\lfloor 0.5-\log_{2}\frac{f_{i}}{f_{\max}}\right\rfloor \;$$

where ⌊..⌋ is the floor function. We denote the frequency of each distinct event *i*, that is, the overall number of occurrences of event *i* within EES, as *f_i_*. We also identify all distinct events for which *f*_max_ = max({*f_i_*: *i* = 1,..,*M*}). As can be concluded from Equation (1), the *FC^IA^* value of the most frequent event is 0, while any event half as frequent as the most frequent event will have *FC^IA^* value equal 1 (due to logarithm with base 2).

For each node that belongs to the ECLG, we find its *FC^IA^* and we rank all the events in ascending order, i.e. from the most to the least frequent event. By setting a threshold for *FC^IA^*, we can remove the least frequent events from the ECLG, i.e. all events with *FC^IA^* larger than this threshold. This allows for extending models with the high confidence, and likely more relevant, events. Additionally, we can keep only the events that co-occur in literature with the most frequent event(s), by removing nodes in ECLG that are not connected to the nodes with *FC^IA^* = 0.

Thus, using the *FC^IA^* metric, we automatically select a subset of EES events to be considered for a model extension, called *candidate extensions*. We will refer to the ECLG obtained automatically after individual assessments and the removal of selected nodes as a *candidate ECLG*.

We note here that, unlike simple naïve event count, the FC metric helps classify events within an EES into several classes, thus allowing modelers to examine events within or across these classes. Moreover, setting a threshold based on a simple event count is arbitrary and does not account for the occurrence frequency of the other events. On the other hand, *FC^IA^* is computed for each event with respect to the most frequent event, allowing modelers to use a threshold and discard the less frequent events.

### 3.2 Pair assessment and clustering

To identify groups of events that would be most useful when added to the model together, we cluster the candidate ECLG with respect to the weights on its edges (literature co-occurrence-based links between events).

#### 3.2.1 Pair assessment (PA)

We measure the co-occurrence of pairs of events within the EES, by computing a frequency class of pairs, *FC^PA^*, and a weighted inverse frequency of pairs, *IF^PA^*. We define the frequency class of a pair of events *i* and *j* within the EES, ${FC}_{i,j}^{PA}$, as:

(2) $${\;FC}_{i,j}^{PA}=\left\lfloor 0.5-\log_{2}\frac{f_{i,j}}{f_{\max,\mathrm{pair}}}\right\rfloor$$

where the co-occurrence frequency of events *i* and *j*, that is, the number of different papers in which both events *i* and *j* occur, is denoted as *f_i, j_*, while $f_{\max,\mathrm{pair}}$ = max({*f_i, j_*: *i* = 1,.,*M*, *j* = 1,.,*M*, *i ≠ j*}).

We also propose an additional pair assessment (PA) metric, ${IF}_{i,j}^{PA}$, that combines the inverse relative frequency of events *i* and *j*, *N*/*f_i_* and *N*/*f_j_*, respectively, where *N* is the total number of events in EES, with a co-occurrence frequency of this pair of events, *f_i, j_*:

(3) $${\;IF}_{i,j}^{PA}=f_{i,j}*\left(\ln\left(\frac{N}{f_{i}}\right)+\ln\left(\frac{N}{f_{j}}\right)\right)\;$$

As can be noticed, the IF^PA^ value increases proportionally to the number of times a pair of events occurs, and it is offset by the sum of the logarithms of the inverse occurrence frequencies of individual events. This inverse factor in the IF^PA^ metric provides several benefits over the FC^PA^ metric, especially in the case of rare but important extracted events. Specifically, using the inverse relative frequency of an interaction, *N*/*f_i_*, increases the likelihood of selecting rare events, and therefore, their impact on the model. The logarithm is used to dampen the effect of the fraction. On the other hand, for frequent events, this fraction is low but still positive.

In order to identify groups of events that would be most useful when added to the model together, we cluster the ECLG using the community detection algorithm proposed by Blondel *et al.* 2008, which has been shown to generate communities of very good quality, outperforming other community detection methods. In the context of the ECLG definition from Section 3.1, given the graph G (V, E, Wv, We), with the node weight set Wv being a set of *FC^IA^* values, and the edge weight set We a set of either *FC^PA^* or *IF^PA^* values, we provide here a brief overview of the community detection algorithm. As defined in Blondel *et al.* (2008), modularity *Q* is a measure of the quality of network partitioning into communities (referred to as clusters in our work) computed as the density of edges inside communities relative to the edges between communities:

(4) $$Q=\frac{1}{2m}\sum_{u,v}\left(w_{u,v}-\frac{\sigma_{u}\sigma_{v}}{2m}\right)*\;\delta \left(c_{u},c_{v}\right)\;$$

where *w_u, v_* represents the weight of an edge between nodes *u* and *v*, *m* is the sum of all edge weights in the network, *c_u_* and *c_v_* are communities of nodes *u* and *v*, $\sigma_{u}$ and $\sigma_{v}$ are sums of the weights of the edges connected to nodes *u* and *v*, respectively; δ(*c_u_*, *c_v_*) = 1, if *u* and *v* belong to the same community, otherwise, it is 0. In order to maximize *Q*, the algorithm has two phases that are repeated iteratively. The first phase starts by assigning each node in the network to its own community, and then, for each node *u*, we compute the change in modularity, ΔQ_u, v_, that would occur if node *u* were to be moved from its current community to the community of each of its neighbor nodes in the network:

(5) $${\Delta Q}_{u,v}=\left[\frac{S_{v}+{2\sigma }_{u,v}}{2m}-{\left(\frac{S_{v,\mathrm{tot}}+\sigma_{u}}{2m}\right)}^{2}\right]-\left[\frac{S_{v}}{2m}-{\left(\frac{S_{v,\mathrm{tot}}}{2m}\right)}^{2}-{\left(\frac{\sigma_{u}}{2m}\right)}^{2}\right]$$

where $S_{v}$ is the sum of weights of the edges inside community *c_v_* that node *u* is moving into, $S_{v,\mathrm{tot}}$ is the sum of the weights of the edges incident to nodes in *c_v_*, σ_*u*_ is the sum of the weights of the edges incident to node *u*, σ_*u, v*_ is the sum of the weights of the edges from node *u* to nodes in *c_v_*, and *m* is the sum of all the weights of all the edges in the network. Once this value is calculated for all communities that *u* is connected to, *u* is placed in the community that resulted in the greatest modularity increase. If no increase is possible, *u* remains in its original community. This process is applied repeatedly for all nodes as long as there is increase in *Q*.

After *Q* reaches a local maximum, the second phase of the algorithm creates a new network where nodes are the communities from the previous phase, the first phase proceeds with this new network, and the iterations are repeated until there is no more increase in *Q*. We will refer to the communities in the undirected candidate ECLG that result from applying this algorithm as *generated clusters*. We show examples of candidate ECLG and the corresponding ECLG with generated clusters in Figure 2.

Next, from the total *N_C_* generated clusters, we are interested in selecting those clusters that would be most useful for extending the model and answer the questions that initiated the literature search. To rank the clusters, we will use the two *PA* literature support metrics. For each cluster C_l_, we find the average values of ${FC}_{i,j}^{PA}$, and ${IF}_{i,j}^{PA}$, across all pairs (*i*, *j*) of connected events *i* and *j* within the cluster C_l_, ${FC}_{l_{\mathrm{avg}}}^{PA}$and, ${IF}_{l_{\mathrm{avg}}}^{PA}$, respectively:

(6) $${\;FC}_{l_{\mathrm{avg}}}^{PA}=\frac{1}{P_{l}}\sum_{\left(i,j\right)}\left({FC}_{i,j}^{PA}\right)$$

(7) $${IF}_{l_{\mathrm{avg}}}^{PA}=\frac{1}{P_{l}}\sum_{\left(i,j\right)}\left({IF}_{i,j}^{PA}\right)\;$$

where *P_l_ =* |E_*l*_| is the total number of edges in cluster C_*l*_.

### 3.3 Interpreted clusters and model support metrics

To add events from generated clusters to a baseline model, we convert the generated clusters, where nodes are events, and edges are literature-based co-occurrences between events, into *interpreted clusters* (weighted directed graphs), with nodes/edges being entities/events.

In addition to ranking the information extracted from literature based on the literature support metrics, we also introduce a model support metric, *Node Overlap* (*NO*), which measures the connectivity of the clusters to the baseline model. More formally, following the definitions from Section 2.2, we denote the baseline model graph as G^BM^(V^BM^, E^BM^), and the graph formed by the EES as G^EES^(V^EES^, E^EES^). For each interpreted cluster $C_{l},$ we can also define $G^{C_{l}}(V^{C_{l}},E^{C_{l}})$, and it is clear from the clustering algorithm that $V^{\mathrm{EES}}=\overset{N_{c}}{\underset{l=1}{\cup }}V^{C_{l}}$. We define the set of *overlapping nodes* between cluster $C_{l}$ and the baseline model as $V^{C_{l},\;\mathrm{ON}}$= $V^{\mathrm{BM}}\;\cap$$V^{C_{l}}$ and the set of *new nodes* in $C_{l}$ as $V^{C_{l},\;\mathrm{new}}$ = $V^{C_{l\backslash }}$($V^{\mathrm{BM}}\;\cap$$V^{C_{l}}$). *NO* is then computed for every interpreted cluster $C_{l}$ to determine the ratio between the overlapping nodes and the total number of nodes:

(8) $${\;NO}_{l}=\frac{\left|V^{C_{l},\;ON}\right|}{\left|V^{C_{l}}\right|}\;\times \;100$$

We also determine whether there are any *return paths* between clusters and the baseline model. Formally, given the definition of an edge in Section 2.2, if there exists a path of connected edges *e^path^*(*v_s_*_1_*, v_tp_*) = [*e_i_*_1_(*v_s_*_1_, *v_t_*_1_), *e_i_*_2_(*v_s_*_2_=*v_t_*_1_, *v_t_*_2_), *e_i3_*(*v_s_*_3_=*v_t_*_2_, *v_t_*_3_), …, *e_ip_*(*v_sp_*=*v_tp_*_-1_*, v_tp_*)], we say that *e^path^*(*v_s_*_1_*, v_tp_*) is a return path, if {*v_s_*_1_*, v_tp_*}∈V^BM^, and all edges *e_i_*_1_, …, *e_ip_* belong to clusters in the set of interpreted clusters. The baseline model and the clusters on such return path form a *candidate extended model.*

### 3.4 Selection of best cluster

We rank all generated clusters with respect to each, average *FC^PA^* and average *IF^PA^*, and their corresponding interpreted clusters with respect to the *NO* value. We also determine which clusters belong to return paths. Finally, we say that cluster $C_{l}$ is assumed to be the best candidate for a model extension if it satisfies the following rule:

(9) $$\left(FC_{l_{\mathrm{avg}}}^{PA}=\min\;\left(\left\{FC_{i_{\mathrm{avg}}}^{PA}:i=1..N_{C}\right\}\right)AND\;IF_{l_{\mathrm{avg}}}^{PA}=\max\left(\left\{IF_{i_{\mathrm{avg}}}^{PA}:i=1..N_{C}\right\}\right)\right)\;\;OR\;\;\left(NO_{l}>50\%\;\mathrm{AND}\;C_{l}\;\mathrm{belongs}\;to\;at\;\mathrm{least}\;\mathrm{one}\;\mathrm{return}\;\mathrm{path}\right)$$

As can be seen from Equation (9), a cluster $C_{l}$ is considered for a model extension if it satisfies either both of the literature support criteria or both of the model support criteria. For the literature support criteria, from Equations (2) and (3), $C_{l}$ must have the lowest ${FC}_{l_{\mathrm{avg}}}^{PA}$value and the highest ${IF}_{l_{\mathrm{avg}}}^{PA}$. This means that the events belonging to $C_{l}$ are the most supported in the literature, among all the events of the EES. On the other hand, for the model support criteria, if the cluster $C_{l}$ has more than a 50% node overlap with the baseline model and it belongs to at least one return path, then $C_{l}$ will be highly connected to the baseline model. Consequently, the cluster $C_{l}$ should be considered for model extension.

We introduced Equation (9) in order to provide a guided and comprehensive way to expand dynamic network models, by adding the events that are not only just frequent in literature but are also connected to the baseline model through return paths.

## 4 Experimental setup

In order to evaluate the performance of CLARINET under different conditions and scenarios, and in the absence of an established and standardized set of benchmarks, we selected three published models that were (i) created and curated manually with input from domain experts, (ii) validated against experimental results, (iii) are of different size and from different contexts and (iv) allow for generating the extension event sets in a different manner. Furthermore, to evaluate how well CLARINET can expand a baseline model, and reconstruct the gold model, we created baseline models of different sizes and with different network structures when compared to the gold models. Using these benchmarks, we demonstrated that CLARINET can assemble relevant and useful models in a *fully automated manner*, starting only with a baseline model and the machine reading output, and *without a knowledge of what is in the gold model*.

### 4.1 Baseline and gold models

**Fig. 3.**
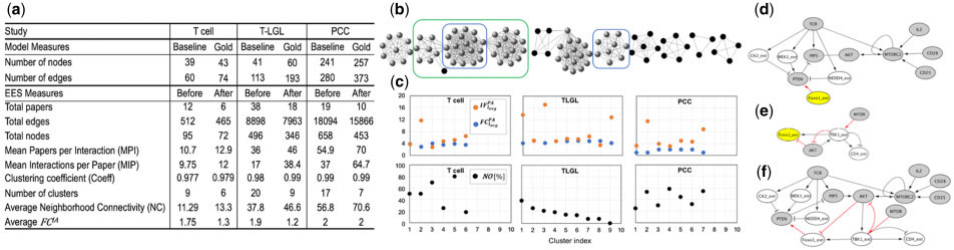
(**a**) Description of each use case in terms of the size of both baseline and gold standard models, followed by the values of several graph metrics for the ECLG before and after the removal of less frequent events, for T-cell, T-LGL and PCC case studies. (**b**) Candidate ECLG for the T-cell case study. (**c**) (Top) Average literature support metrics ${\mathrm{FC}}_{\mathrm{avg}}^{\mathrm{PA}}$ and ${\mathrm{IF}}_{\mathrm{avg}}^{\mathrm{PA}}$ for generated clusters. (Bottom) Node overlap (NO) between the clusters and the baseline model for the three case studies. (**d–f**) Cluster interpretation and preparation for extension: (d), (e) Interpreted clusters’ influence graphs, C_3_, C_2_, respectively, for T-cell case study, nodes are biological entities, pointed arrows represent activation, blunt arrows represent inhibition. Baseline model nodes are in gray and the new nodes with suffix ‘_ext’ are in white. (d) Cluster C_3_ with upstream element Foxo1_ext highlighted in yellow, (e) cluster C_2_ with downstream element Foxo1_ext. The return path from C_2_ to C_3_ is (‘MTOR—TBK1_ext—AKT– Foxo1_ext—PTEN’), highlighted in red, the first node and last node of the path are MTOR and PTEN, respectively, (f) the result of merging C_3_ and C_2_

Here, we detail the steps of creating baseline models in our three case studies. The network characteristics of all studied model networks are listed in Figure 3a, and a brief background for the three modeled systems is provided in the Supplementary Material.

The first benchmark gold model that we used is the naïve T-cell differentiation (T-cell) model from Hawse *et al.* (2015) (Tcell^gold^). In Miskov-Zivanov *et al.* (2013), the authors present a manually created logical model of the naïve T-cell differentiation that recapitulated key experimental observations and generated several predictions, and we will use this model as the baseline model (Tcell^baseline^) for this study. The model in Hawse *et al.* (2015) is a manually extended version of the original model from Miskov-Zivanov *et al.* (2013). Tcell^baseline^ has 9% less nodes and 18% less edges when compared to Tcell^gold^.

Our second benchmark gold model is a manually created discrete dynamic T-cell large granular lymphocyte (T-LGL) leukemia model from Zhang *et al.* (2008) (TLGL^gold^). This model was used in Saadatpour *et al.* (2011) to perform a comprehensive dynamical and structural analysis, which in turn led to identifying 19 model elements as potential therapeutic targets. We created the baseline model for this study (TLGL^baseline^) by removing all direct regulators of these 19 key players in T-LGL, which resulted in a significantly smaller number of nodes and edges in the baseline model network, compared to the TLGL^gold^ model, i.e. 32% less nodes and 41% less edges (Fig. 3a).

The third benchmark gold model that we used is the pancreatic cancer cell (PCC) model from Telmer *et al.* (2019) (PCC^gold^), a discrete manually created model of the major signaling pathways, metabolism and gene regulation, and accounting for the tumor microenvironment. The PCC model was used in Telmer *et al.* (2019) to explore pancreatic cancer receptor stimulation and mutation response in time through simulations. This model describes the hallmarks of cancer (processes of apoptosis, autophagy, cell cycle progression, inflammation, immune response, oxidative phosphorylation and proliferation) and suggests combinations of inhibitors as therapies. Based on evidence from literature (Wang *et al.*, 2016), mTORC1 initiates autophagy, TGFβ regulates apoptosis and KRAS mutations enhance proliferation. Thus, to evaluate how well CLARINET can reconstruct critical signaling pathways, we created the baseline model for this study (PCC^baseline^) by removing the paths between mTORC1 and autophagy, between TGFβ and apoptosis, and between KRAS and proliferation. This resulted in the removal of 6% nodes and 25% edges from the original PCC^gold^ model (see Fig. 3a).

### 4.2 Extracted event sets

In this work, we use an open-source reading engine REACH (Valenzuela-Escárcega *et al.*, 2017) to quickly obtain information from the literature. REACH is available online and can also be run through INDRA (Gyori *et al.*, 2017). To create the EES for the T-cell study, among 32 references cited by (Hawse *et al.*, 2015), we selected 12 most relevant papers, that is, papers in which T cell is mentioned together with one or more of the key elements of the Tcell^baseline^ model from Miskov-Zivanov *et al.* (2013). We then used REACH to extract events from these papers. The size of the EES for this and the other two studies is reported in Figure 3a under column ‘Before’. We used a similar approach when creating the EES for the PCC study, having REACH engine read 19 papers cited in Telmer *et al.* (2019), as those papers provided evidence for the manually constructed PCC model in Telmer *et al.* (2019). However, as the rows ‘Total nodes’ and ‘Total edges’ in Figure 3a show, the EES obtained for the PCC study was much larger than the EES for the T-cell study.

We used a different approach when assembling the EES for the T-LGL study. Instead of relying on the same literature that was used to manually build the published gold standard model TLGL^*gold*^, we created a search query ‘T cell large granular lymphocyte (T-LGL) leukemia and proliferation and apoptosis’, and we used it as an input to the literature search engine [PubMed (Roberts, 2001)]. From the papers that PubMed returned, we selected the 38 papers that PubMed identified as ‘Best match’. The final step was the same as with the other two studies, the EES is extracted using REACH. Interestingly, while an order of magnitude larger than the EES for the T-cell study, the EES obtained using a search query was smaller than the one we obtained for the PCC study, emphasizing the fact that the number of events found within a single paper can vary significantly.

## 5 Results

To evaluate CLARINET and demonstrate its features, we conducted several experiments using the three models described in Section 4. We explored how well CLARINET performs in various scenarios, small versus large model extension, controlled versus query-based extension and extension of a smaller published model versus reconstruction of a truncated model.

### 5.1 Model extension with CLARINET

While CLARINET is fully automated, and parametrizable, to demonstrate its flexibility and the outcomes of parametrizations, we also show here results for intermediate steps.

For each baseline model and EES, CLARINET creates an ECLG (as described in Section 3.1), similar to the one shown in Figure 3b (T-cell). For all nodes (events) in the original ECLG, CLARINET then computes *FC^IA^* according to Equation (1). As stated previously, events with *FC^IA^* = 0 are the most frequent ones, thus strongly supported by literature, with multiple evidence statements. The users can enter a value for the *FC^IA^* as a threshold for removing less frequent events (i.e. events found less often in the selected set of papers); otherwise, CLARINET assumes the average *FC^IA^* value within the EES as a default threshold. We found that an average value in all three case studies is *FC^IA^* = 2, and using this default threshold, we removed events with *FC^IA^* > 2 from the ECLG. In Section 5.3, we will discuss the influence of this threshold on the performance of CLARINET. Using *FC^IA^* = 2, CLARINET removed 20, 150 and 205 less frequent events from EES in the T-cell, T-LGL and PCC studies, respectively.

In Figure 3b, we highlight with black color the nodes that are being removed from the ECLG for our T-cell study. The number of nodes and edges in the ECLG before and after this step is shown in Figure 3a. As can be noticed, after the removal of the less frequent nodes, not only the size of the ECLG changed but also other graph parameters changed. For instance, the mean number of papers per interaction, which maps to the average degree of nodes (events), increased after the removal of the less frequent nodes. The removal led to a denser graph, with strongly connected components, which is in agreement with both the increased neighborhood connectivity of the nodes and the high clustering coefficient.

CLARINET can use an additional selection criterion (Section 3.1), to keep only the nodes of the reduced ECLG that are neighbors of the nodes representing most frequent (*FC^IA^* = 0) events, and it removes the rest of the nodes from the reduced ECLG. In other words, CLARINET can remove events that do not co-occur with any of the most frequent events. In Figure 3b, the subgraph enclosed in a green box is an ECLG that we would obtain for the T-cell study if we applied this additional selection criterion.

Next, after applying the literature support metrics and obtaining the candidate ECLG, following the method from Section 3.2, CLARINET assigns weights to all edges in the candidate ECLG, using two different sets of weights, *FC^PA^* and *IF^PA^*, for two separate clustering procedures. CLARINET partitioned the candidate ECLG into six, nine and seven edge-weighted generated clusters, for T cell, T-LGL and PCC, respectively. These clusters include interactions from 6, 18 and 10 out of the 12, 38 and 19 papers that were selected at the beginning (Fig. 3a). Using the two different metrics (*FC^PA^* and *IF^PA^*) to weigh edges did not affect the number of generated clusters and the edges within each cluster for our three studies; however, while the *IF^PA^* values had a larger discrepancy between clusters, the *FC^PA^* values seem to be much closer to one other.

To select the best-generated cluster(s) that would be most useful when added to the baseline model, following Equations (6) and (7), CLARINET computes for each cluster the average *FC^PA^* and *IF^PA^* values and ranks the clusters according to these values. Since the *FC^PA^* and *IF^PA^* values computed for any given edge are usually different, the clusters’ average *FC^PA^* and *IF^PA^* values are also different, and therefore, the ranking of clusters with respect to these values can differ as well. For each case study, we show the average *FC^PA^* and *IF^PA^* values for generated clusters in Figure 3c. As can be noticed, for the T-cell case, the ranking of clusters from lowest to highest average *FC^PA^* value is C_2_, C_6_, C_4_, C_1_, C_5_, C_3_, and the ranking of clusters from highest to lowest average *IF^PA^* value is C_2_, C_6_, C_5_, C_4_, C_1_, C_3_. From these rankings, we see that cluster C_2_ is suggested as the best cluster in both cases, that is, it has the lowest average *FC^PA^* value, and the highest average *IF^PA^* value among all six clusters. On the other hand, in the T-LGL case study (Fig. 3c), the *FC^PA^*-based ranking is C_3_, C_1_, C_9_, C_7_, C_5_, C_4,_ C_6_, C_2_, C_8_, whereas the *IF^PA^*-based ranking is C_3_, C_1_, C_9_, C_7_, C_5_, C_6_, C_4_, C_2_, C_8_. For the PCC model, the corresponding cluster rankings are C_2_, C_7_, C_1_, C_4_, C_5_, C_6_, C_3_ and C_2_, C_7_, C_5_, C_6_, C_3_, C_1_, C_4_, respectively.

Next, CLARINET transforms these generated clusters into interpreted clusters and explores the connection between the interpreted clusters and the baseline model. Figure 3c shows the *NO* values [equation (8)], for the clusters of each case study. In the T-cell case study, clusters C_3_ and C_5_ have the highest *NO* value, i.e. the highest percentage overlap with the baseline model. For T-LGL, clusters C_1_ and C_2_ are the ones with the highest *NO*, whereas for PCC, clusters C_2_, C_4_ and C_7_ all have high *NO* values. We can conclude from these results that the *NO* measure identifies different clusters, compared to the ones with the highest *FC^PA^* and *IF^PA^* weights. This demonstrates the versatility of CLARINET and the flexibility it provides to users in choosing different strategies for automated model extension.

In addition to the metrics discussed above, the user may also be interested in extending a baseline model to include a particular element and to study its effects on the model. In such cases, if there are two or more clusters in the set of interpreted clusters that we obtained, all containing regulators and regulated elements of the element of interest, CLARINET can instead consider those clusters for extension. We are especially interested in combining these clusters if they can be connected through a return path, which starts and ends in the model, as defined in Section 3.3. If the user selects this option, CLARINET can find return paths, thus enabling users to add key regulatory pathways that are not in the baseline model.

To illustrate the return paths, the set of events that are included in the top ECLG cluster enclosed by the blue box in Figure 3b, is also shown as interactions in interpreted cluster C_3_ in Figure 3d. It can be seen from Figure 3d that Foxo1_ext, which is a new element in the EES, is activating PTEN, which is also an element in the baseline model, Tcell^baseline^. If we add only the cluster from Figure 3d to Tcell^baseline^, we will be able to study the effect of Foxo1_ext, as it will become an input to Tcell^baseline^. However, with such extension, we will not be able to study the effect of the other parts of the model on Foxo1_ext, given that Foxo1_ext is not regulated by any other element in Tcell^baseline^. Therefore, CLARINET can search for other clusters that include Foxo1_ext regulators. One such cluster is C_2_ (Fig. 3e).

Cluster C_2_ also corresponds to the bottom cluster enclosed by a blue circle in Figure 3b. In the set of the six interpreted clusters that CLARINET obtained for our T-cell model case study, clusters C_2_ and C_3_ form a return path with Tcell^baseline^, as shown in Figure 3f. Thus, the final set of events that CLARINET formed by merging the two clusters in Figure 3f, contains all the elements of the full model Tcell^gold^ from Hawse *et al.* (2015) (FOXO1, NEDD4, MEK1, CK2), which were missing in Tcell^baseline^ from Miskov-Zivanov *et al.* (2013).

Similarly, for T-LGL case study, in the set of the nine interpreted clusters that CLARINET obtained, clusters C_3_ and C_9_ form a return path with the baseline model, TLGL^baseline^. Therefore, CLARINET provides the set of events formed by merging clusters C_3_ and C_9_ as our finally selected set of TLGL^baseline^ extensions. Finally, for the PCC case study, in addition to the return path that CLARINET found between clusters C_2_, C_7_, and the baseline model, PCC^baseline^, making the union of these two clusters a good candidate for extension, CLARINET also found return paths between an individual cluster C_2_ and PCC^baseline^. Similar to the T-cell study, CLARINET was also able to closely reproduce TLGL^gold^ and PCC^gold^ models published in Zhang *et al.* (2008) and Telmer *et al.* (2019), as further detailed in the following.

### 5.2 Precision and recall

To evaluate the relevance and the completeness of the entities and events that CLARINET selects, we computed its precision and recall. This was done by comparing the final models, Tcell^*final*^, TLGL^*final*^ and PCC^*final*^, that were obtained using CLARINET, with the gold standard models, Tcell^gold^ (Hawse *et al.*, 2015), TLGL^gold^ (Zhang *et al.*, 2008) and PCC^gold^ (Telmer *et al.*, 2019), respectively (see Supplementary Fig. S1).

**Fig. 4.**
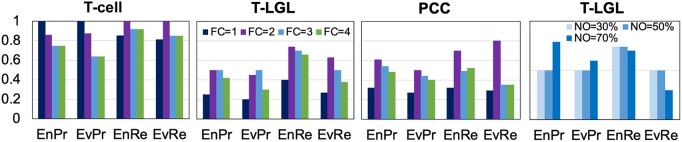
Precision and recall of CLARINET when compared to the gold standard model for T-cell, T-LGL and PCC use cases. EnPr, EvPr, EnRe and EvRe denote entity precision, event precision, entity recall and event recall, respectively

The precision value indicates the relevance by determining the percent of events (or entities) that are selected by CLARINET, and which are at the same time a part of the gold standard model. These are usually called true positives, and therefore, we will refer to these entities and events as *true* entities and events. In Figure 4, we show precision results for all three final models (*FC^IA^* = 2), for both entities and events. For the T-cell case, CLARINET achieves high precision for both events and entities, 0.86 and 0.87, respectively. This means that just 14% of the events and 13% of the entities that CLARINET selected are false positives (i.e. they are not in the gold standard model). On the other hand, for the T-LGL case, the event precision is 0.45 and the entity precision is 0.5, and in the PCC case, it is 0.61 and 0.5, respectively. While in the T-LGL and PCC studies almost half of the events and entities are false positives, it is important to note that these two studies have much larger EES, compared to the T-cell study and also compared to their baseline models, and thus, have more candidates to add to the model. Additionally, the events that are in the gold standard models are not necessarily the only valid events. In other words, there could be other events in literature, and which CLARINET suggested in its output, that are also useful and important, and should be included in the model. Therefore, the precision of CLARINET that we report here is likely smaller than its actual precision due to these additional important events that CLARINET finds but are not in the gold standard model.

To investigate this further, we conducted the following exercise for the T-LGL study. We used INDRA (Gyori *et al.*, 2017) to compute a belief score for each event that CLARINET selected. Interestingly, we found that INDRA generated a belief score with a value greater than or equal to 0.7 (out of 1) for 27 events and 21 entities, not all of which were in the CLARINET’s true event and true entity sets. When we changed the status of these additional entities and events from false positives to true positives, this has increased the entity precision to 0.7 and event precision to 0.64. Moreover, these events form more than one return path with the baseline model, i.e. they are highly connected to the baseline model. Additionally, if preferable, one can reduce the number of false positives by increasing the threshold for the *NO* value, as will be discussed in the following subsection.

To evaluate the completeness of CLARINET results, we computed its recall with respect to the gold standard models. We will refer to all entities and events in gold standard models as *correct* entities and events. We compute recall as the ratio between true events (or entities) selected by CLARINET and the total number of correct events (or entities) found in the EES. We note here that we only account for those events from the gold standard model that is in the EES, as it is possible that there are events in the gold standard model that are not in the reading output and in the baseline model, and therefore, not present in CLARINET’s input. This is due to the reading engine not recognizing in papers all the events, while the human reader who cited the papers was able to find the events and manually include them in the model.

For the T-cell study, a recall value of 1 has been reported for both events and entities. This means that none of the correct events or entities are missed by CLARINET, that is, there are zero false negatives. For PCC case, the event recall value is 0.8 and the entity recall value is 0.7. Here, CLARINET achieved better values for recall than for precision. This again demonstrates the ability of CLARINET in identifying the useful and relevant entities and events in a given EES. Similarly, in the T-LGL case recall values are higher than precision values. As shown in the figure, the entity recall is 0.74 and the event recall of 0.63, i.e. CLARINET missed approximately 26% of correct entities and 37% of correct events. The slightly lower recall in this study, when compared to the T-cell and PCC studies, is not surprising. Given the significant portion of events (41%) removed from the TLGL^gold^ model to obtain TLGL^baseline^, and the literature co-occurrence criteria, it is not surprising that CLARINET found in the large EES many additional entities and events that have stronger connections with the baseline model than the ones that are in the gold model.

### 5.3 Parameter selection

We explored the effect on precision and recall when varying the two key parameters from Equation (9), the thresholds for *FC^IA^* and *NO* values. Varying the *FC^IA^* threshold, affected the size of candidate ECLG. Consequently, this affected the number and the size of generated clusters. Increasing the *FC^IA^* threshold, i.e. including more of the less frequent events in the analysis, increases the size of the candidate ECLG and will increase the number of generated clusters (Supplementary Table S1). As can be seen from Figure 4, the best results were obtained for *FC^IA^* threshold of 2 for T-cell and PCC cases. For T-LGL, the *FC^IA^* threshold of 2 or 3 resulted in similar precision and recall, while the threshold equal to 2 had a better event recall value.

Overall, the *FC^IA^* threshold of 2 achieved the best results for all cases. As a reminder, this value is the average *FC^IA^* value that we obtained for the EES of each case, and the user can choose to specify this threshold value as an input to CLARINET. It is also important to keep in mind that the low *FC^IA^* threshold results in selecting the most frequent nodes, with the expense of ignoring any infrequent nodes that may be of interest, and the high *FC^IA^* threshold leads to larger number of clusters and longer runtimes, without much benefit.

In Figure 4, we also show the effect of three different thresholds for *NO* on precision and recall. As can be noticed, any *NO* threshold below 50%, will not affect the precision and recall values. Increasing this *NO* parameter to a value higher than 50% will ensure a more connected cluster to the model, and thus, fewer false positives. However, this may not be a desirable solution in the cases when we are interested in identifying other entities and events that are not necessarily in the model. Our analysis suggests that an *NO* value of 50% or more, along with finding return paths, is sufficient in determining how well a cluster is connected to the model, while not missing potentially useful new information from the literature.

### 5.4 Scalability

We also investigated how scalable CLARINET is when applied on models and EES with different sizes. We have run and tested CLARINET on a 3.3 GHz Intel Core i5 processor. We have found that, for the T-cell case, having both small model and small EES (see Fig. 3a), CLARINET took 2.5 s to run and generate clusters. For the T-LGL study, with only a slightly bigger model, but a large EES, CLARINET took 10.1 s. And finally, for the PCC case, when we applied CLARINET on a large model and a much larger EES compared to the previous two cases, the runtime was 25.4 s. Therefore, CLARINET can very efficiently extend baseline models that already have several hundred nodes, while exploring candidates from an EES with tens of thousands of events.

### 5.5 Comparison with other extension methods

To evaluate our new automated extension method against previously presented work (Liang *et al.*, 2017; Sayed *et al.*, 2018a), we applied each method on the EES that we obtained in the T-cell model case study, and we compared the selected groups of extensions. We show the selected candidate extensions obtained with the methods (Liang *et al.*, 2017) and (Sayed *et al.*, 2018a) in Supplementary Figure S2a and b, respectively. The extension method proposed in Liang *et al.* (2017) adds candidate extensions to the baseline model in layers. For example, all candidate extensions with both nodes in the baseline model belong to layer 0, those with one node in the baseline model are in layer 1, and so on. Therefore, the output of the method proposed in Liang *et al.* (2017) includes elements that do not regulate other model elements (thus, called ‘hanging’), and they can be seen in Supplementary Figure S2a. This makes their methodology less practical, especially if it is applied on a large-scale model and large EES. Compared to the work in Liang *et al.* (2017), CLARINET approaches the model extension challenge in a more inclusive way, by combining several metrics, based on occurrence and co-occurrence frequencies in published literature, and the connectivity to the baseline model. This way, CLARINET provides groups of connected events that are also well connected with the baseline model through return paths (Fig. 3f).

We also show in Supplementary Figure S2b, several groups of extensions selected by (Sayed *et al.*, 2018a). Due to the nondeterministic behavior of the genetic algorithm, there is more than one set of extensions selected with this method. However, all the selected subsets share the same characteristics, they contain several disconnected components, and they lack the main regulations for PTEN, which is one of the key elements in Tcell^gold^ (Hawse *et al.*, 2015). Interestingly, the extensions are connected through a return path, AKT→Foxo1_ext→PTEN, which means the interactions are connected to Tcell^baseline^. However, when compared to the manually extended model, there are still some missing interactions such as CK2→PTEN, MEK1→PTEN and NEDD4→PTEN. Moreover, similar to the results obtained for the method from Liang *et al.* (2017), there are several candidate extensions that include hanging nodes, and therefore, do not affect the model.

As shown in Supplementary Figure S3, the genetic algorithm-based (Sayed *et al.*, 2018a) method achieves a better entity and event precision than the method in Liang *et al.* (2017). The low event precision 0.3, and low entity precision 0.21 in Liang *et al.* (2017), is due to the large number of false positives. On the other hand, the entity and event precision of the genetic algorithm-based method are 0.57 and 0.53, respectively. Both methods have similar entity recall values of approximately 0.8 in Liang *et al.* (2017) and 0.7 in Sayed *et al.* (2018a), however, the genetic algorithm-based method missed a number of events, resulting in a low event recall of 0.4, whereas the event recall value of Liang *et al.* (2017) is 0.8. On the other hand, as shown in Supplementary Figure S3, CLARINET outperformed the results in Liang *et al.* (2017) and Sayed *et al.* (2018a). For the T-cell case study, entity and event precision values are 0.87 and 0.86, respectively. Moreover, the recall value is 1 for both entities and events.

Thus, from the comparisons, we conducted for this case study, using the literature and model support metrics, CLARINET outperforms the methods from Liang *et al.* (2017) and Sayed *et al.* (2018a) in selecting the best set of model extensions.

## 6 Conclusion

We presented here our tool, CLARINET, and its underlying methodology that integrates information from published literature and expert-built models to rapidly assemble or extend models. CLARINET is parametrizable, it allows users to select different extension criteria, depending on the context, focus and goals of their models. By automatically extending models with the information published in the literature, our methodology allows for rapid collection of the existing information in a consistent and comprehensive way, while facilitating information reuse and data reproducibility, and replacing hundreds or thousands of manual experiments, thereby reducing the time needed for the advancement of knowledge. We tested CLARINET on three previously published biological networks of different sizes with different machine reading outputs that varied in size from hundreds to tens of thousands. CLARINET was able to reproduce these manually built networks with an average recall of 0.8, while also identifying new interactions with high confidence, all within several seconds.

## Funding

This work was supported in part by the AIMCancer award from the Defense Advanced Research Project Agency (W911NF-17-1-0135).

*Conflict of Interest*: none declared.

## Supplementary Material

vbab006_Supplementary_Data
