# Supplementary material for: CLARINET: efficient learning of dynamic network models from literature
Source: Bioinform Adv. 2021 Jun 3;1(1):vbab006. doi: 10.1093/bioadv/vbab006 (PMC9710628; doi:10.1093/bioadv/vbab006)
Supplement: vbab006_Supplementary_Data [file vbab006_supplementary_data.zip › CLARINET_supplement_editable.docx]

*Bioinformatics*, YYYY, 0–0

doi: 10.1093/bioinformatics/xxxxx

Advance Access Publication Date: DD Month YYYY

Manuscript Category

| Subject Section  **CLARINET: Efficient learning of dynamic net-work models from literature**  Yasmine Ahmed^1,*^, Cheryl Telmer^2^ and Natasa Miskov-Zivanov^1,3,*^  ^1^Electrical and Computer Engineering Department, ^3^Bioengineering Department, Computational and Systems Biology Department, University of Pittsburgh, ^2^Department of Biological Sciences, Carnegie Mellon University, Pittsburgh, PA, USA.  *To whom correspondence should be addressed.  Associate Editor: XXXXXXX  Received on XXXXX; revised on XXXXX; accepted on XXXXX |
| --- |

# Modeled systems

## Naïve T cell differentiation

Naïve T cells are stimulated via antigen presentation to the T cell receptor (TCR) and with co-stimulation at CD28 receptor. This stimulation results in the activation of several downstream pathways, forming feedback and feedforward loops between pathway elements, which then leads to the differentiation of naïve T cells into either the helper (Th) or the regulatory (Treg) phenotype. The distribution between Th and Treg cells within the T cell population depends on antigen dose; high antigen dose results in mostly Th cells, while low antigen dose leads to a mixed population of Th, Treg, and undifferentiated cells [1]. The key markers that are commonly used to measure the outcomes of the naïve T cell differentiation are IL-2 and Foxp3, where Th (Treg) cells are characterized by the high (low) expression of IL-2 and low (high) expression of Foxp3.

## T cell large granular lymphocyte (T-LGL) leukemia

The T cell large granular lymphocyte (T-LGL) leukemia is a disease characterized by an abnormal increase of cytotoxic T lymphocytes (CTLs) [2]. As described by authors in [2], similar to normal activated CTL, leukemic T-LGL exhibit activation of multiple survival signaling pathways. Unlike normal activated CTL, leukemic T-LGL are not sensitive to Fas-induced apoptosis, a process essential for activation-induced cell death. There is no curative therapy yet known for this disease. Hence, there is a crucial need to identify potential therapeutic targets. A discrete dynamic model has been proposed in [2] to understand the signaling components that determine the survival of CTL cells in T-LGL leukemia. The model incorporates the signaling pathways involved in normal cytotoxic T cells activation and the known deregulations of survival signaling in leukemic T-LGL. The model includes proteins, mRNAs, small molecules such as lipids and biological processes that indicate cell fate such as cytoskeleton signaling, proliferation and apoptosis.


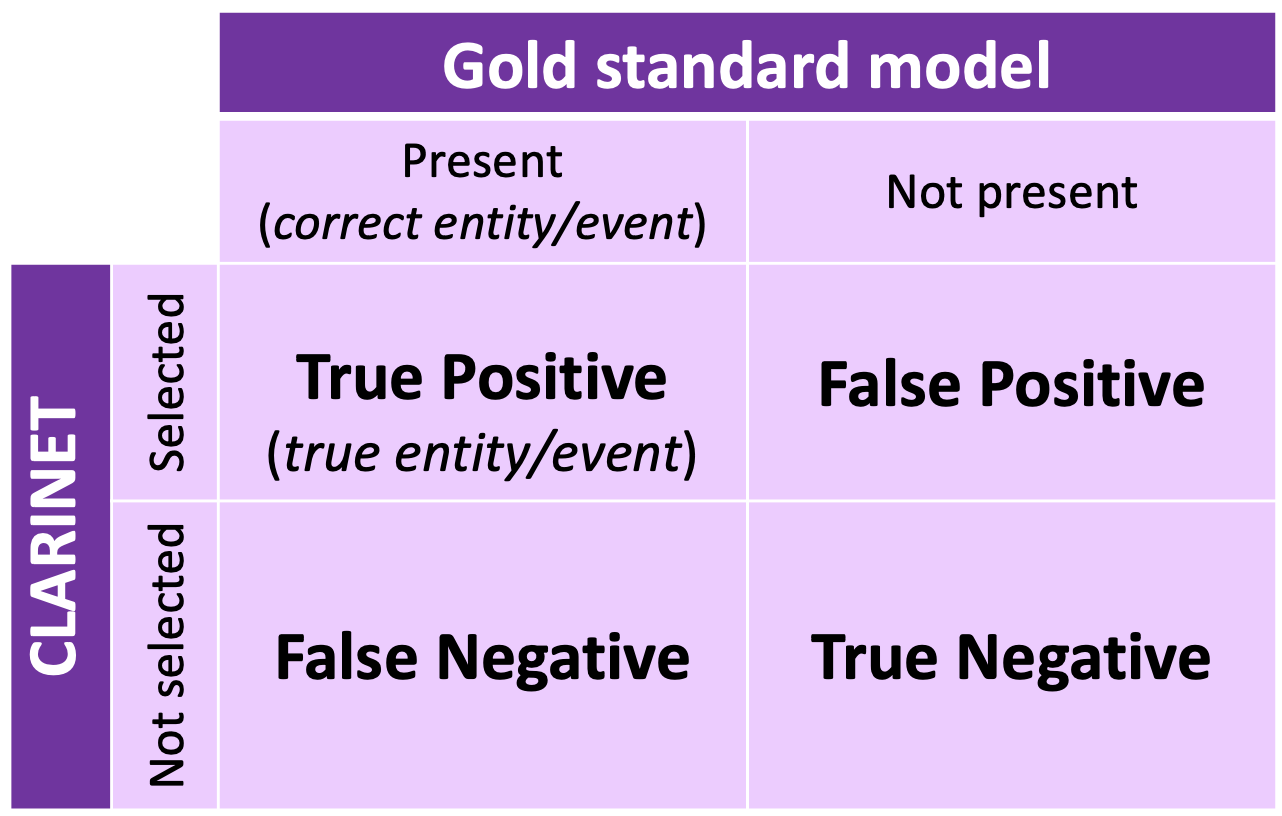


**Fig. S1. The matrix used to compute precision and recall of CLARINET.**

## Pancreatic cancer microenvironment

Although some cancers such as breast and colon can be managed, others such as glioma and pancreatic cancer have very poor survival rates [3]. As described in [3], pancreatic cancer has early KRas activating mutations followed by TP53 and CDN2A inactivating mutations in the majority of tumors. Although the pancreatic cancer has a known mutational profile disrupting signaling pathways, there are no available drugs to target KRas activation or restore tumor suppressor function, and therefore, survival of patients has not improved. The modeling of pancreatic cancer is of great importance since it could reveal molecular mechanisms important for disease treatment. Therefore, the focus of such model is to include the major signaling pathways, metabolism and the tumor microenvironment. The pancreatic cancer model proposed in [3] describes the hallmarks of cancer (which are represented as the processes of apoptosis, autophagy, cell cycle progression, inflammation, immune response, oxidative phosphorylation and proliferation) and suggests combinations of inhibitors as therapies.

**Fig. S3. Precision and recall when compared to gold standard model for T cell use case, for methods in [4], [5] and CLARINET. EnPr, EvPr, EnRe and EvRe denote entity precision, event precision, entity recall and event recall, respectively.**


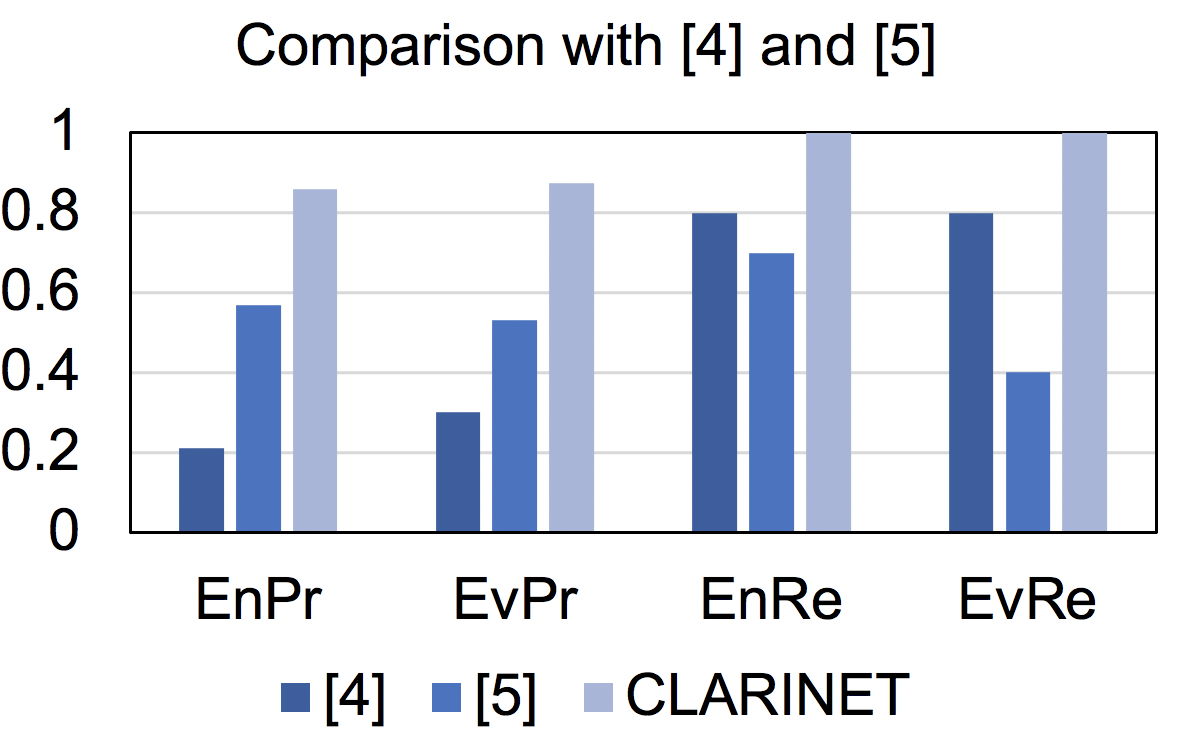


**Fig. S2. Comparison between the finally selected interactions by (a) Liang et al. work [4], (b) The genetic algorithm-based method [5].**


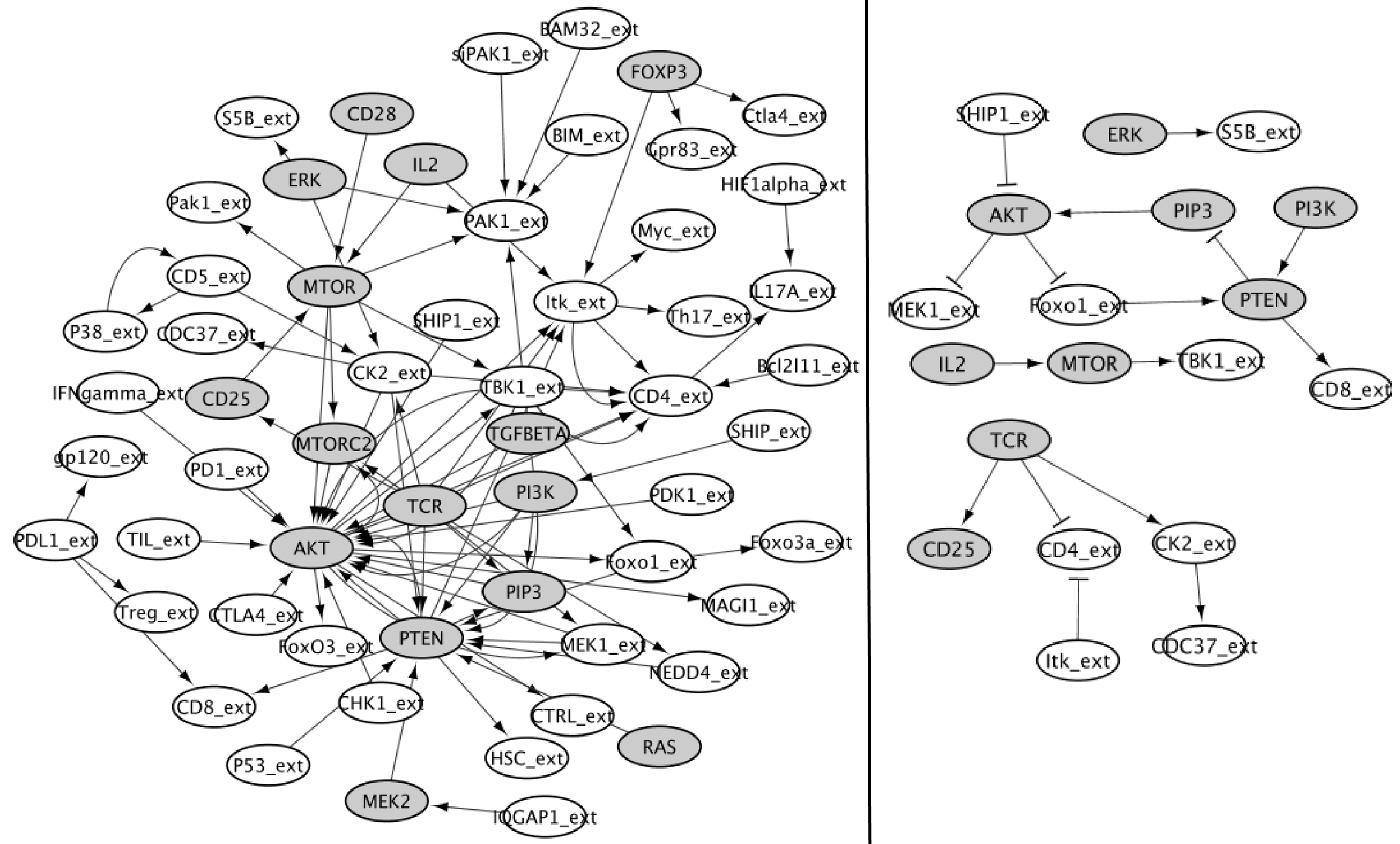


1. (b)


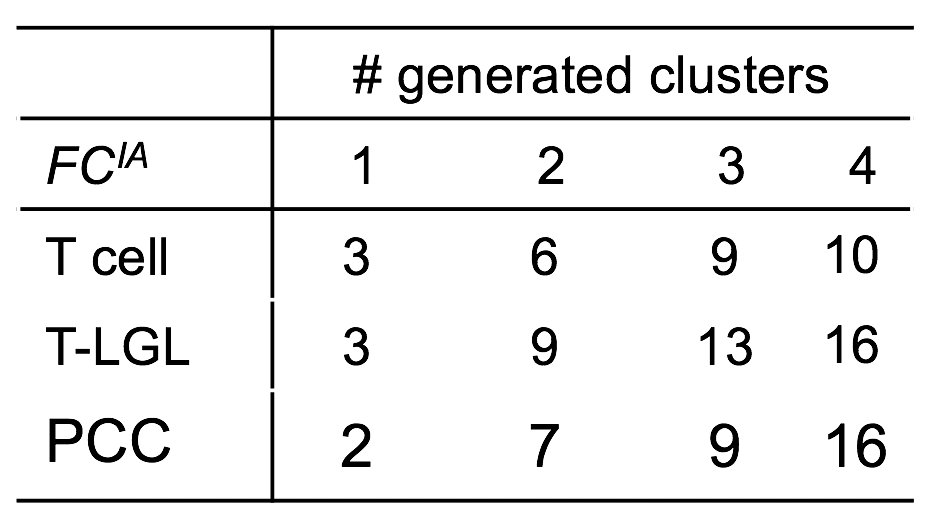


**Table S1. Number of generated clusters for T cell, T-LGL and PCC use cases using different frequency class values.**

References

[1] N. Miskov-Zivanov, M. S. Turner, L. P. Kane, P. A. Morel, and J. R. Faeder, “The duration of T cell stimulation is a critical determinant of cell fate and plasticity,” Sci. Signal., vol. 6, no. 300, pp. 1–16, 2013.

[2] R. Zhang et al., “Network model of survival signaling in T-cell large granular lymphocyte leukemia,” Proc. Natl. Acad. Sci., vol. 105, no. 42, pp. 16308–16313, 2008.

[3] C. Telmer et al., “Computational modeling of cell signaling and mutations in pancreatic cancer,” in AI for Synthetic Biology, 2019.

[4] K.-W. Liang, Q. Wang, C. Telmer, D. Ravichandran, P. Spirtes, and N. Miskov-Zivanov, “Methods to Expand Cell signaling Models using Automated Reading and Model Checking.” Springer, Cham, pp. 145–159, 2017

[5] K. Sayed, K. N. Bocan, and N. Miskov-zivanov, “Automated Extension of Cell Signaling Models with Genetic Algorithm,” no. 1, pp. 5030–5033, 2018.
