# Supplementary figures and images for: CLARINET: efficient learning of dynamic network models from literature
Source: Bioinform Adv. 2021 Jun 3;1(1):vbab006. doi: 10.1093/bioadv/vbab006 (PMC9710628; doi:10.1093/bioadv/vbab006)

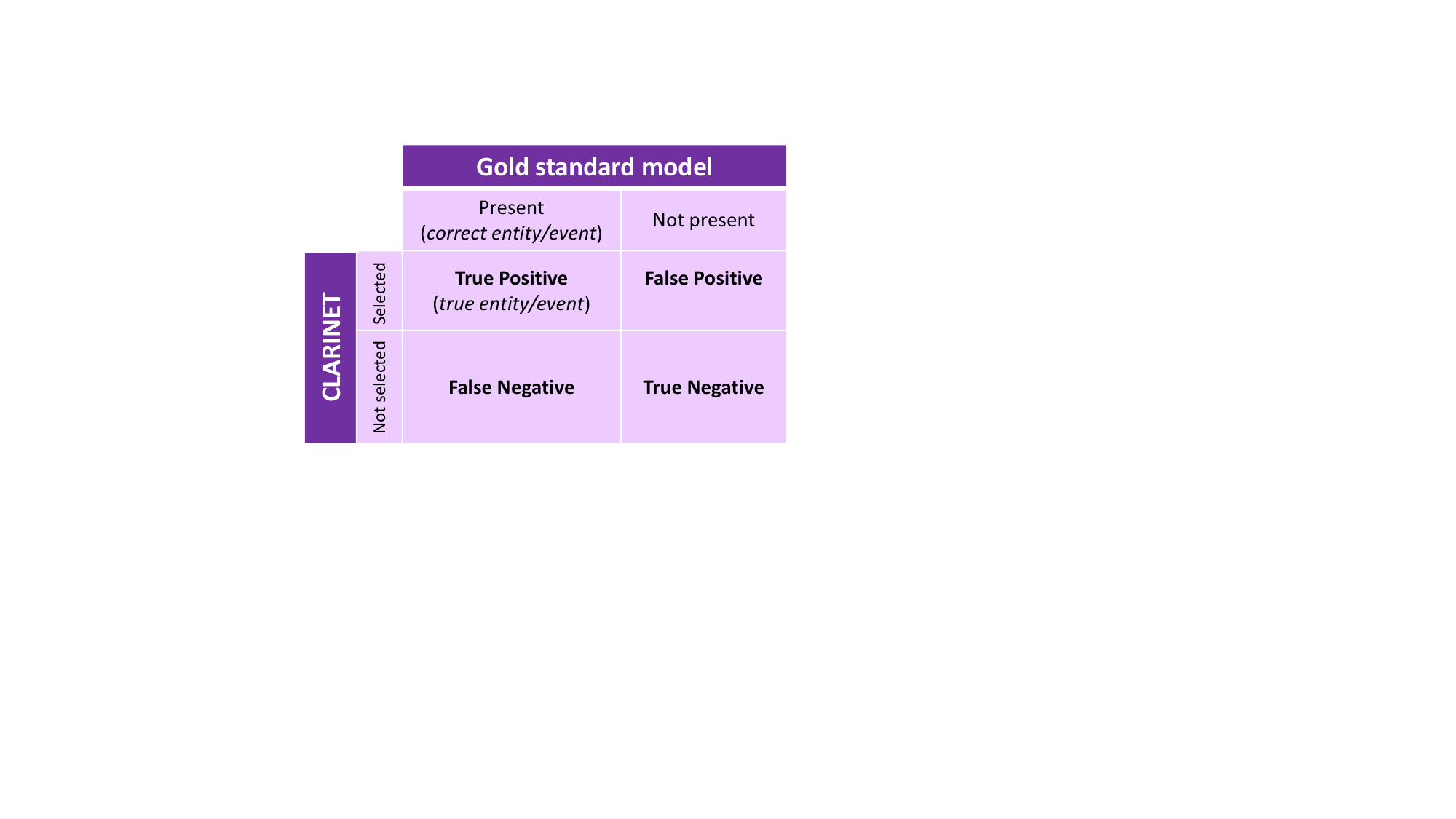

Supplement: vbab006_Supplementary_Data [file vbab006_supplementary_data.zip › Fig.S1.tiff]

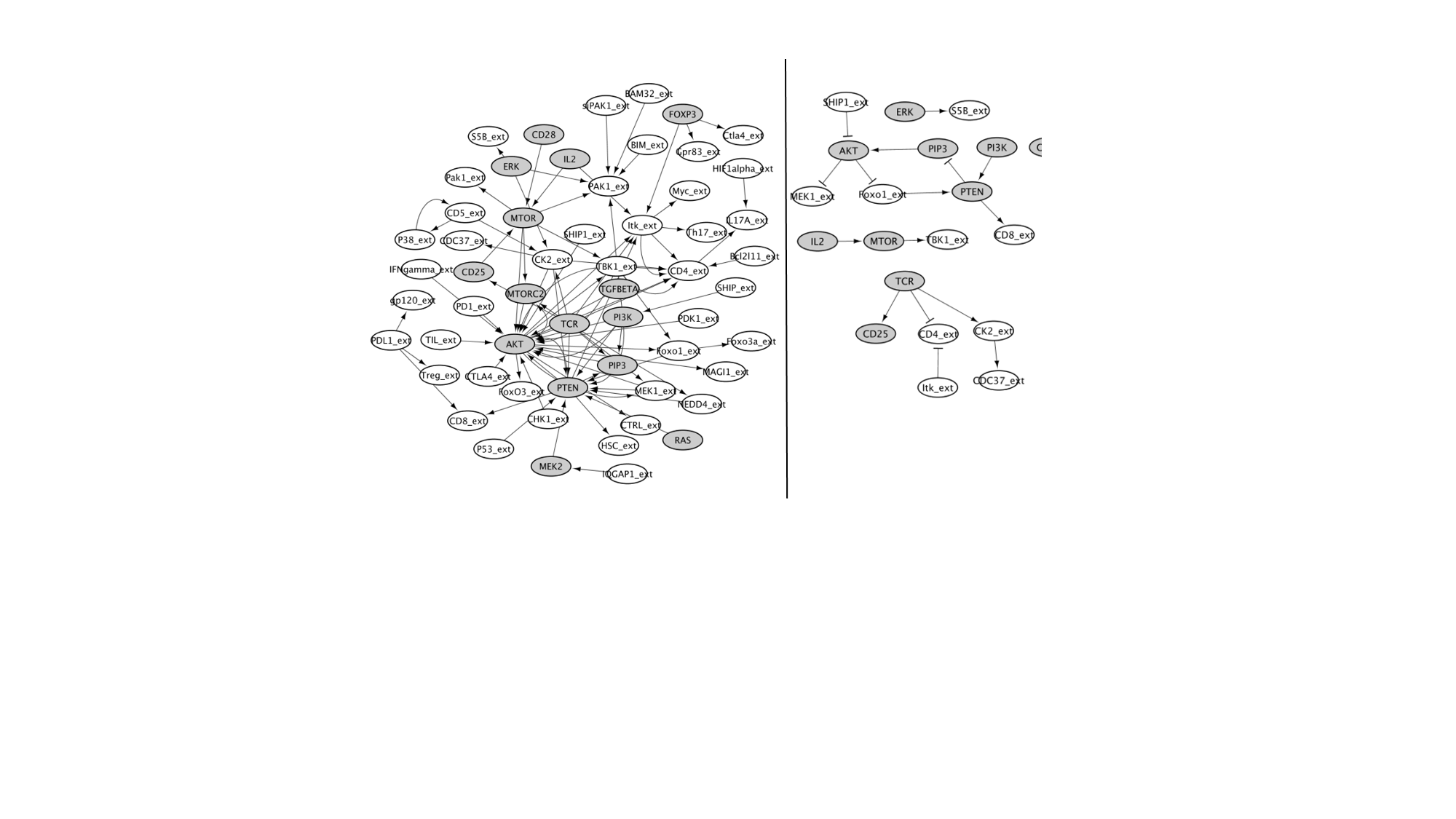

Supplement: vbab006_Supplementary_Data [file vbab006_supplementary_data.zip › Fig.S2.tiff]

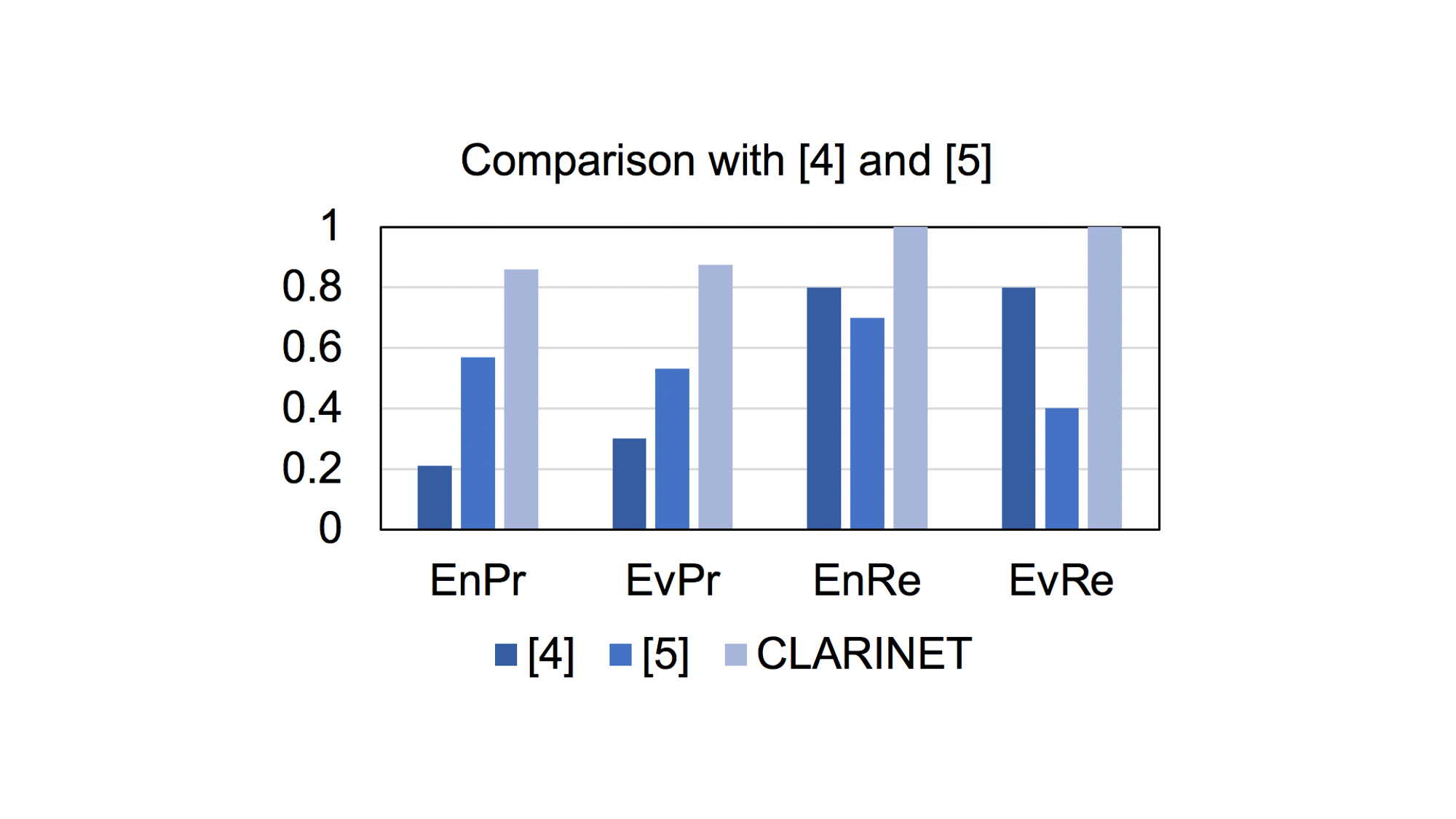

Supplement: vbab006_Supplementary_Data [file vbab006_supplementary_data.zip › Fig.S3.tiff]

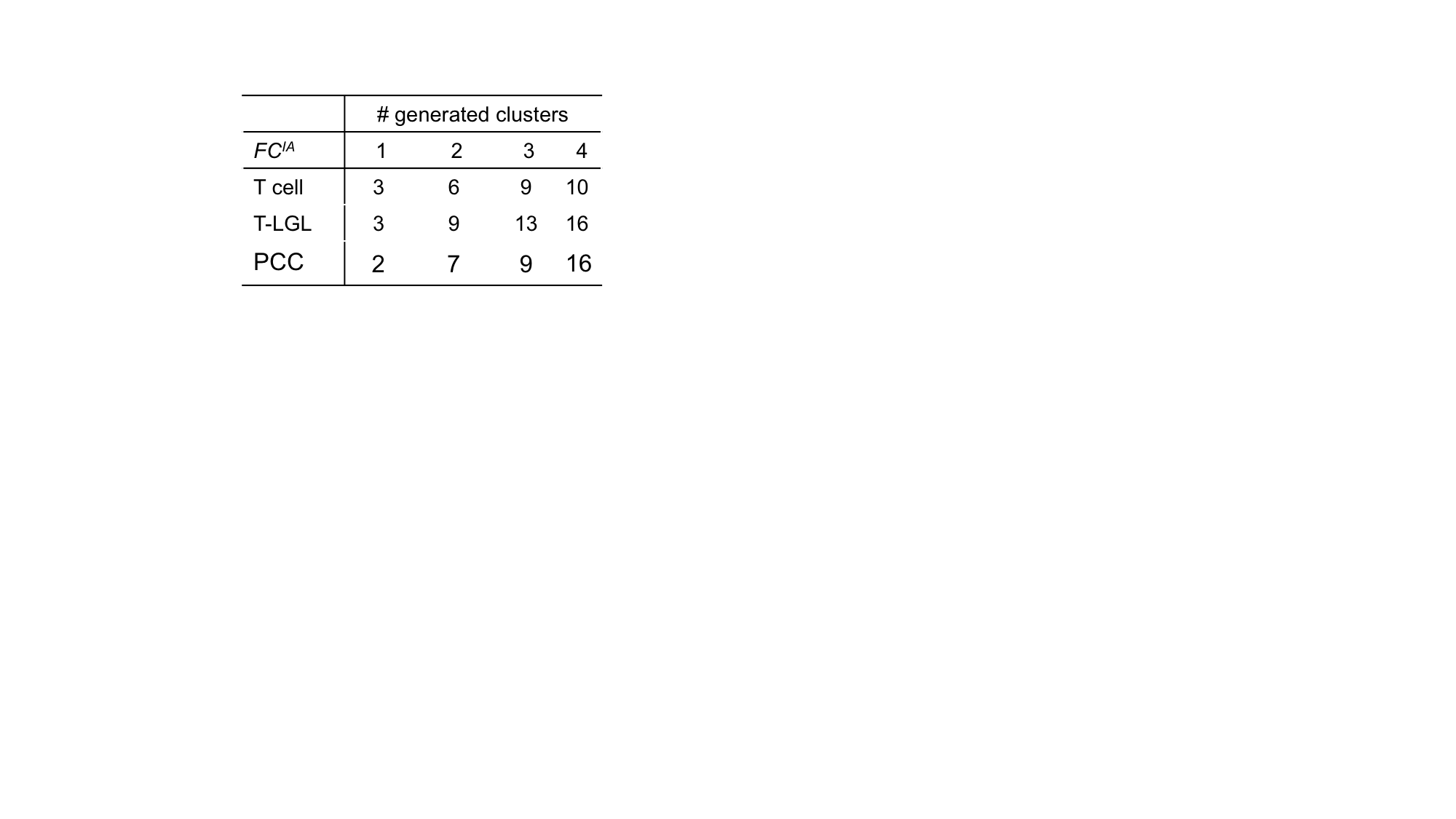

Supplement: vbab006_Supplementary_Data [file vbab006_supplementary_data.zip › Table S1.tiff]
